# Metalloproteinases ADAM10 and ADAM17 Mediate Migration and Differentiation in Glioblastoma Sphere-Forming Cells

**DOI:** 10.1007/s12035-016-0053-6

**Published:** 2016-08-19

**Authors:** Elodie J. Siney, Alexander Holden, Elizabeth Casselden, Harry Bulstrode, Gareth J. Thomas, Sandrine Willaime-Morawek

**Affiliations:** 1 Clinical Neurosciences, Faculty of Medicine, University of Southampton, Southampton, SO16 6YD UK; 2 Wessex Neurological Centre, University Hospital Southampton, Southampton, SO16 6YD UK; 3 Cancer Sciences Unit, Faculty of Medicine, University of Southampton, Southampton, SO16 6YD UK; 4 Southampton General Hospital, LF51, South Laboratory Block, Southampton, SO16 6YD UK

**Keywords:** Glioma, Cancer stem cell, Cell migration, Cell differentiation, Extracellular matrix, Disintegrin

## Abstract

Glioblastoma is the most common form of primary malignant brain tumour. These tumours are highly proliferative and infiltrative resulting in a median patient survival of only 14 months from diagnosis. The current treatment regimens are ineffective against the small population of cancer stem cells residing in the tumourigenic niche; however, a new therapeutic approach could involve the removal of these cells from the microenvironment that maintains the cancer stem cell phenotype. We have isolated multipotent sphere-forming cells from human high grade glioma (glioma sphere-forming cells (GSCs)) to investigate the adhesive and migratory properties of these cells in vitro. We have focused on the role of two closely related metalloproteinases ADAM10 and ADAM17 due to their high expression in glioblastoma and GSCs and their ability to activate cytokines and growth factors. Here, we report that ADAM10 and ADAM17 inhibition selectively increases GSC, but not neural stem cell, migration and that the migrated GSCs exhibit a differentiated phenotype. We also observed a correlation between nestin, a stem/progenitor marker, and fibronectin, an extracellular matrix protein, expression in high grade glioma tissues. GSCs adherence on fibronectin is mediated by α5β1 integrin, where fibronectin further promotes GSC migration and is an effective candidate for in vivo cancer stem cell migration out of the tumourigenic niche. Our results suggest that therapies against ADAM10 and ADAM17 may promote cancer stem cell migration away from the tumourigenic niche resulting in a differentiated phenotype that is more susceptible to treatment.

## Introduction

In recent years, there have been significant advances in cancer therapy; survival rates for haematological and breast cancers, for example, have improved dramatically, yet for the primary brain tumour glioblastoma, median survival rate remains at 14 months from diagnosis.

This is partly due to the aggressive nature of these tumours which are highly proliferative and invasive. First line treatments of surgical resection, chemo- and radiotherapy fail to prevent tumour recurrence within months giving weight to the theory proposed by Singh and colleagues [1] that glioblastoma is fuelled by cancer stem cells (CSCs) [2]. CSCs have similar characteristics to neural stem cells (NSCs), but instead of producing properly differentiated neural cell types, they produce anaplastic clones that form the tumour mass.

A primary goal for glioblastoma research would be selective ablation of the CSC compartments; but in the absence of a unique and highly specific marker for CSCs in glioblastoma, this is not yet possible. Another approach would be to target the nature of these CSCs and to alter or inhibit properties that make them stem like and tumourigenic by in situ cell differentiation to inhibit stem cell properties from CSCs [3].

The role of the niche in tumour biology is increasingly recognised with CSCs requiring an environment that supports growth and maintains expression of genes necessary for self-renewal. Expression of chemokines induces migration of CSCs to the niche [4], and this process also requires degradation of the extracellular matrix (ECM) by proteinases. We, along with others, have previously identified overexpression of the metalloproteinases ADAM10 and ADAM17 in glioblastoma [5–7], where increased ADAM10 or ADAM17 expression correlates with poorer prognosis [8, 9]. These closely related proteins are capable of growth factor and cytokine processing; ADAM17 specifically cleaves TNFα (tumour necrosis factor alpha) [10] a mediator of inflammation and tumour initiation and potentiation [11], and ADAM10 sheds epidermal growth factor (EGF) [12] a key player in cell proliferation and survival whose receptor is mutated in approximately 50 % of glioblastoma cases. ADAM10 and ADAM17 are also important during development for glial cell migration and can influence cell differentiation through cleavage of Notch [13, 14].

Previous reports using commercial glioblastoma cell lines suggest that ADAM10 and ADAM17 inhibition decrease tumour growth and invasiveness [15, 16] but these do not specifically address the behaviour of the tumourigenic cells. Here, we elucidate the specific role that these two metalloproteinases play in high grade glioma sphere-forming cell (GSC) migration and differentiation. By isolating enriched stem cell populations from human glioblastoma and inhibiting these two proteins in in vitro cell migration models, we found for the first time that ADAM10 and ADAM17 inhibition increased migration in GSCs but not NSCs and that the migrated cells are more differentiated compared to non-migrated cells. Migration being linked to adhesion, we showed that GSC adherence on fibronectin is mediated by α5β1 integrin, where fibronectin further promotes GSC migration and is an effective candidate for in vivo cancer stem cell migration out of the tumourigenic niche. Our new results suggest that ADAM10 and ADAM17 may be involved in retaining GSCs in the tumourigenic niche in vivo.

## Materials and Methods

### Sample Collection and Cell Culture

Excised tumour from 12 high grade glioma patients (Table 1) was collected into artificial CSF on ice then micro-dissected to remove necrotic regions and major blood vessels. Remaining tissue was digested in artificial cerebral spinal fluid (ACSF) containing hylauronidase, kinurenic acid and trypsin. Cells were plated at 100 cells/μl in complete media: NeuroCult™ + supplement (StemCell Technologies Ltd. #05702), 1 % antibiotics/antimycotic, human fibroblastic growth factor-basic, (bFGF 10 ng/ml PeproTech #100-18B), human epidermal growth factor (EGF 20 ng/ml PeproTech #100–15) and heparin (2 μg/ml Sigma #3149) in 25-cm^2^ vented plastic flasks pre-coated with 25 μg/ml laminin (Sigma #L2020) and maintained at 37 °C, 5 % CO_2_. At 24 h, the media was removed and discarded and remaining adherent cells were transferred into fresh flasks without laminin to allow clonal sphere formation. Samples were maintained at 37 °C, 5 % CO_2_ and passaged at least three times before assaying.

**Table 1:** Tissue samples and patient characteristics

Samples	Sex	Age at time of resection	WHO grade	Tumuor type	GFAP staining	Ki67 staining
G002	F	69	IV	Glioblastoma	Positive	Moderate to high
G036	F	37	III	Anaplastic oligoastrocytoma	Positive	Moderate
G037	M	62	IV	Glioblastoma	Positive	High
G049	F	72	IV	Glioblastoma	Positive	Moderate
G065	M	57	IV	Glioblastoma	Positive	High
G071	F	47	III	Anaplastic oligodendroglioma	Positive	High
G083	M	37	IV	Glioblastoma	Positive	High
G097	M	70	IV	Glioblastoma	Variable	High
G099	M	34	IV	Glioblastoma with oligodendroglial component	Positive	High
G100	M	73	IV	Glioblastoma	Positive	High
G109	F	64	IV	Glioblastoma	Positive	Moderate to high
G112	M	67	IV	Glioblastoma	Positive	High

### Inhibitors

To inhibit ADAM10 and ADAM17 in vitro, we used antibodies against hADAM10 (Millipore #AB19026) and hADAM17 (Calbiochem #PC491) and the non-specific ADAM17 inhibitor TAPI-2 (Peptides International #INH-3852-PI). Human recombinant ADAM17 (Calbiochem #PF133) was used as a positive control. To screen integrin subunit interactions, we used the α integrin blocking kit (Millipore #ECM430), anti-β1 integrin (Abcam #24693) and anti-β6 integrin [17] (gift from Gareth Thomas).

### Sphere Formation

GSC spheres were dissociated and passed through a 40-μm filter prior to adding into a 12-well plate at 5 × 10^3^ cells per well. Wells contained complete culture media with or without blocking antibodies. At day 11, spheres >100 μm diameter were counted with the average of triplicate wells being reported.

### Monolayer Cell Culture

GSC spheres were dissociated and passed through a 40-μm filter prior to adding into a 48-well plate pre-coated with 25 μg/ml laminin (Sigma #L2020), human fibronectin (FN, R&D systems #1918-FN-02M) or human vitronectin (VN, R&D systems #2349-VN-100) at 5 × 10^3^ cells per well in complete media. These cultures were maintained at 37 °C, 5 % CO_2_ for 14 days with a 50 % media change at days 5 and 10.

### Immunofluorescence

Excised tumours were collected into ACSF on ice; tissues were fixed in 4 % paraformaldehyde, cryoprotected and sectioned on a cryostat; cells were fixed in 4 % paraformaldehyde. Tissues and cells were permeabilised with 0.2 % Triton X-100 in phosphate-buffered saline (PBS). Non-specific binding was blocked by 10 % donkey serum in permeabilisation buffer. Cells and tissues were incubated with primary antibodies: anti-hADAM10 (Millipore #AB19026), anti-hADAM17 (Calbiochem #PC491), anti-S100beta (Abcam #ab52642), anti-laminin (Sigma #L9393), anti-fibronectin (AbdSerotec #4470–4339), anti-vitronectin (Abcam #ab13413) and anti-beta1 integrin (Abcam #ab24693) all at 1:100, anti-nestin (Abcam #ab28944), anti-Sox2 (Y17) (Santa-Cruz #sc-17320) and CD133/2 (Miltenyi Biotech #130-090-851) all at 1:200 and anti-beta-III-tubulin (Covance #mms-435p) at 1:500; and secondary antibodies are the following: anti-rabbit Alexa Fluor 488 or anti-mouse Alexa Fluor 568 at 1:200 (Life Technologies). 4'6-diamidino-2-phenylindole (DAPI) was used for nuclear visualisation. Images were taken using a fluorescent Leica DM IRBE microscope or a Leica SP5 laser scanning confocal microscope, as indicated.

### Enzyme Activity Test

ADAM17 protease activity of human recombinant ADAM17 (200 ng/ml Calbiochem #PF133) or G002 cell lysates (1 × 10^5^ cells per well) were tested for their ability to cleave the fluorescent substrate, MCA-KPLGL-Dpa-AR-NH2 using the InnoZyme TACE activity kit (Calbiochem #CBA042). Cells were lysed using a buffer containing 1 % NP-40 and no protease inhibitors. Cell lysates were incubated on ice for 30 min with ADAM17 inhibitors; anti-ADAM17, TAPI-2 or control IgG at (10 μg/ml) prior to following the manufacturer’s protocol.

### Transwell Migration Assays

Lower chambers were pre-incubated with or without attractants for 1 h at 37 °C then blocked with 0.1 % bovine serum albumin (BSA) in serum-free media without growth factors for 30 min. 5 × 10^4^ cells were then added to the upper transwell® chamber (Corning #3422). Transwell plates were incubated at 37 °C, 5 % CO_2_ for 24 h. Cells of the lower chambers were collected and counted using trypan blue on a haemocytometer.

### Sphere Spreading

Six to eight individual spheres (100 μm diameter) were placed onto pre-coated wells with 25 μg/ml laminin (Sigma #L2020), human fibronectin (FN, R&D systems #1918-FN-02 M) or human vitronectin (VN, R&D systems #2349-VN-100), containing complete media, 1 μM cytosine β-*D*-arabinofuranoside (Ara-C, Sigma #C1768) and either 2 μg/ml IgG isotype control (Cambridge Biosciences #400101), 0.2 μg/ml anti-ADAM10 (Millipore #AB19026), 2 μg/ml anti-ADAM17 (Calbiochem #PC491) or anti-ADAM10 and ADAM17 combined. Spheres were imaged from 0 to 45 h; sphere area was calculated using Image J freehand tool.

### Cell Adhesion Assays

96-well flat bottom plates were coated with fibronectin (25 μg/ml) or 0.1 % BSA (non-specific adhesion control) for 1 h at 37 °C then blocked with 0.1 % BSA for 30 min. Cells were dissociated, washed in serum free media, passed through a 40-μm filter then incubated on ice with or without inhibitors for 30 min. 2 × 10^4^ cells were added to each well containing serum free media +0.1 % BSA and incubated at 37 °C, 5 % CO_2_ until non-specific binding occurred. Non-adhered cells were removed by washing with PBS; adhered cells were fixed with 1 % glutaraldehyde for 10 min then stained with 0.02 % crystal violet. Cell adherence was calculated by colorimetric analysis using Varioskan™ Flash (Thermo Scientific) measuring absorbance at 585 nm and subtracting non-specific adhesion values.

### Flow Cytometry

Cells were collected and permeabilised in PBS + 1 % Tween-20 for 5–10 min; non-specific binding was blocked with PBS + 0.01 % BSA. Cells were incubated with fluorophore-conjugated antibodies: anti-human βIII-tubulin-Alexa Fluor647 (BDbiosciences #560394), anti-human nestin Alexa Fluor488 (eBiosciences #53-9843-82), anti-humanCD133/2-PE (Miltenyi Biotech #130-090-853) all at 1:333. Samples were run on FACSCanto I (BD Biosciences) and analysed using the FACSDiva software.

### Statistics

All statistical analyses were performed using Prism v.6 (GraphPad, Inc.) unless otherwise stated. Experiments were carried out at a minimal of triplicate. Error bars indicate SEM unless otherwise stated and significance results were reported for *P* < 0.05.

## Results

**Fig. 1:**
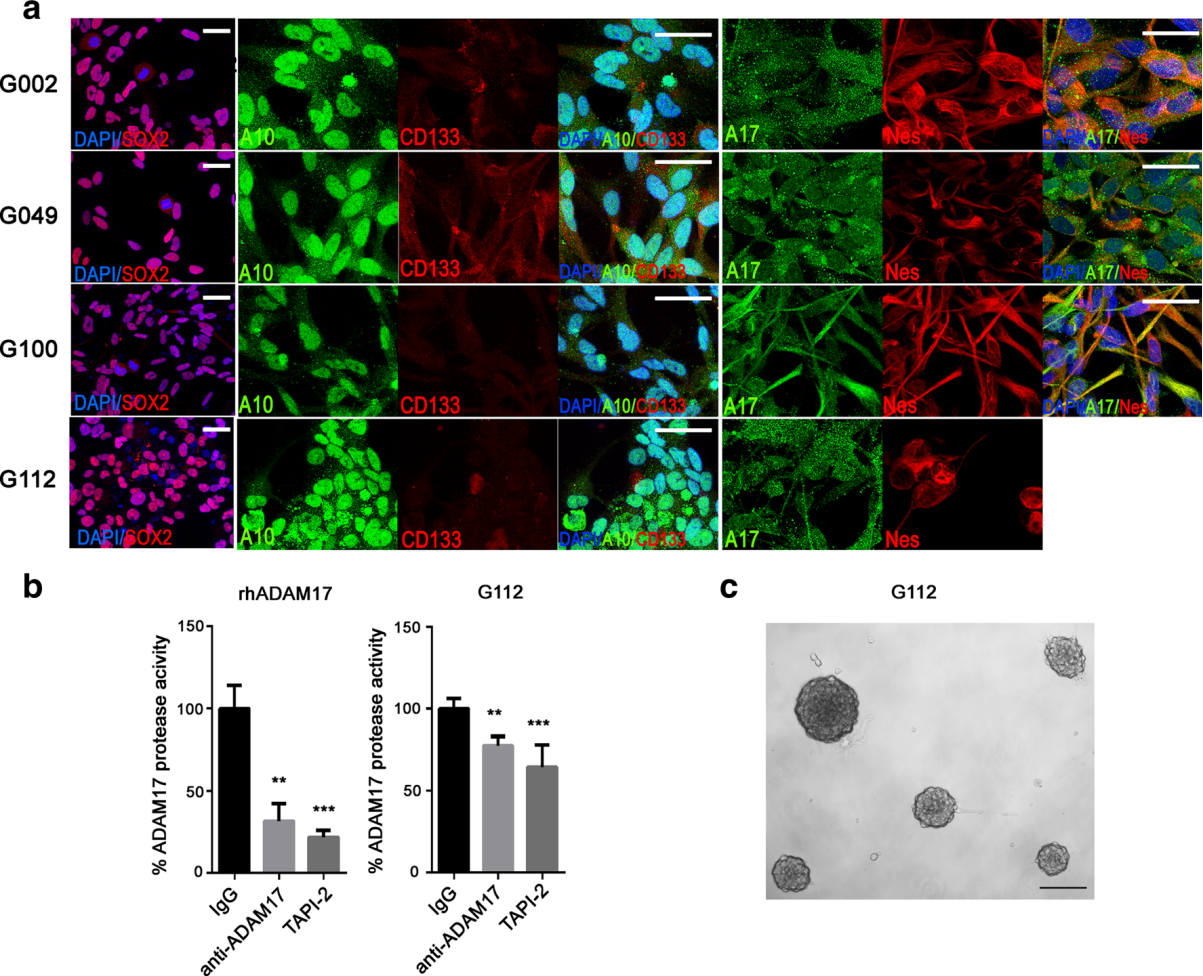
ADAM10 and ADAM17 are expressed in patient-derived GSCs and anti-ADAM17 inhibits protease function. **a** Representative confocal images of metalloproteinases ADAM10 (A10, *green*) and ADAM17 (A17, *green*) and stem cell markers SOX2 (*red*), CD133 (*red*) and nestin (Nes; *red*) staining of GSCs cultured as monolayers from four cell lines (G002, G049, G100 and G112). Nuclei stained with DAPI (*blue*), *scale bar* = 20 μm. N.B. G112 ADAM17 and nestin staining are from different wells, hence the absence of a merge picture. **b** The protease activity of purified rhADAM17 (*left*) or G002 cell lysates (*right*) pre-treated with either IgG isotype control (10 μg/ml), anti-ADAM17 (10 μg/ml) or TAPI-2 (10 μg/ml). Two separate G002 cell lysates were collected at passages 3 and 5, respectively, and tested in triplicate; the purified rhADAM17 was tested once in triplicate. **P < 0.01, ***P < 0.001 compared to IgG control using one-way ANOVA followed by Tukey’s posthoc test. **c** Tumourspheres from G112 after 11 days in vitro, *scale bar* = 100 μm

Using a first adherent culture step on laminin [18], we have isolated a population of cells from glioblastoma tissues cultured in serum-free conditions with mitogens to favour the maintenance of stem/progenitor cells. These cultures exhibit stem cell properties; they are capable of self-renewal over 20+ passages and can differentiate into immature neurons and astrocytes [5]. Although not a homogenous stem cell population, these cultures reliably produce clonal spheres after dissociation (Fig. 1c) and are therefore termed glioma sphere-forming cells (GSCs).

### ADAM10 and ADAM17 Inhibition Increases GSC, but not NSC, Directional Migration

Confirming previous results [19], we show that ADAM10 and ADAM17 are expressed in GSCs (Fig. 1a) along with colocalisation of ADAM10 and ADAM17 and nestin [5]; moreover, we have previously reported increased mRNA levels in both glioblastoma tissues and corresponding cultured spheres established from some of the same patient samples used in the present study (including G002, G037, G049). To validate the use of blocking antibodies, we demonstrated the ability of specific ADAM10 and ADAM17 antibodies to inhibit the protease function of ADAM17 as a purified protein [5] and we now show the ability of these antibodies to inhibit the protease function of ADAM17 in GSC lysates from G002, at levels comparable to the metalloproteinase inhibitor TAPI-2 (Fig. 1b).

**Fig. 2:**
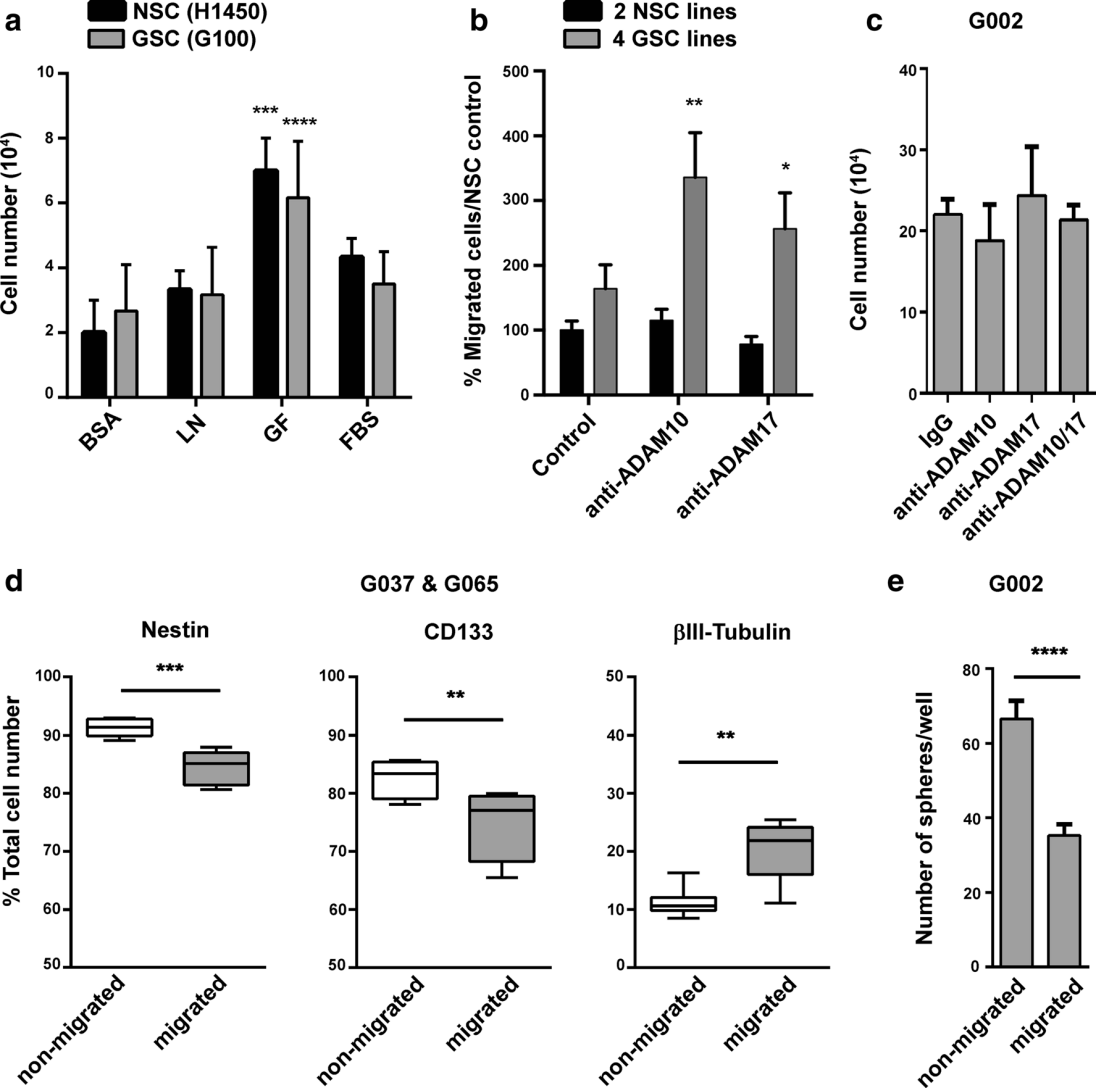
ADAM10 and ADAM17 inhibition increases GSC, but not NSC; directional migration and the migrated GSCs are more differentiated. **a** Migration of neural stem cells from foetal sample H1450 (NSC) and isolated GSCs from patient sample G100 (GSC) towards selected chemoattractants. Lower transwell chambers contained bovine serum albumin (BSA, 0.1 g/ml), laminin (LN, 10 μg/ml), growth factors (GF: EGF 20 ng/ml, FGF 10 ng/ml and heparin 2 μg/ml) or foetal bovine serum (FBS, 0.1 %). *N* = 3 wells per condition and per cell line; ***P < 0.001 and ****P < 0.0001 compared to BSA using two-way ANOVA followed by Tukey’s posthoc test. **b** Chemoattraction of two NSC (H1445 and H1450) and four GSC (G002, G036, G037 & G049) lines with either anti-ADAM10 (0.2 μg/ml), anti-ADAM17 (2 μg/ml) or no treatment (control) in lower chamber. Cell numbers in lower chambers were normalised to the NSC control to give percent cell migration. *N* = 4 wells per condition and per cell line; *P < 0.05, **P < 0.01 compared to the control using two-way ANOVA followed by Bonferroni’s posthoc test. **c** 24-h toxicity test of ADAM10 and ADAM17 blocking antibodies on GSCs from G002. 2.5 × 10^5^ cells were added into wells containing IgG (2 μg/ml), anti-ADAM10 (0.2 μg/ml) anti-ADAM17 (2 μg/ml) or anti-ADAM10 and anti-ADAM17 combined. Cell viability was calculated using trypan blue to visualise and discount dead cells. *N* = 3 wells per condition; *P* = 0.8292 using one-way ANOVA. **d** Percentage of migrated and non-migrated GSCs expressing stem cell/progenitor marker nestin, putative brain tumour stem cell marker CD133 or neuronal marker βIII-tubulin from two GSC lines (G037 and G065). *N* = 2 wells per condition and per cell line; **P < 0.01, ***P < 0.001 using the Mann-Whitney test. **e** Sphere formation ability of migrated vs. non-migrated GSCs, from patient sample G002, following transwell migration assay. 5 × 10^3^ cells from the upper (non-migrated) and lower (migrated) chambers were plated in triplicate. At day 11, spheres >100 μm were counted and averaged. ****P < 0.0001 using unpaired *t* test

As cells usually migrate in response to environmental cues, we decided to find a suitable attractant for directional migration by adding attractants into the lower chambers in transwell assays. The combination of mitogens used in culture (EGF, FGF and heparin) was the most effective stimulus for both GSCs and NSCs (Fig. 2a). This was thus used to test the effect of ADAM10 and ADAM17 inhibition on cell migration (Fig. 2b), as we checked that over the 24-h assay period, there was no significant change in cell numbers when treated with the ADAM blocking antibodies (Fig. 2c). In four GSC lines, migration increased twofold in the presence of anti-ADAM10 and 1.6-fold with anti-ADAM17 (Fig. 2b) but there was no significant change in the number of NSCs migrating into the lower transwell chamber towards the mitogens. Combined ADAM10 and ADAM17 inhibition decreases cell proliferation after 3 days [5], and here, we found no effect on cell death over 24 h on the same cell line (G002). Hence, the increase in number of cells in the migration assay in four GSC lines is not due to changes in cell proliferation or cell death but specifically to directional migration of GSCs. We thus show here that inhibition of ADAM10 and ADAM17 increases migration of GSCs.

### Migrated GSCs Are More Differentiated than Non-Migrated GSCs

Next, we compared the expression of stem cell and differentiation markers in migrated vs. non- migrated cells. By separating the two populations at 24 h in the transwell assay, we found decreased expression of nestin and CD133 in the migrated population from GSC lines along with increased expression of βIII-tubulin (Fig. 2d). The sphere formation potential of these two populations was then assessed; the migrated population showed a 50 % reduction in the number of spheres produced compared to non-migrated cells (Fig. 2e). There was no significant differences in the size of the spheres from migrated and non-migrated cells (data not shown), thus excluding an effect of proliferation on this experiment. This demonstrates on three lines that migrated cells are more differentiated than non-migrated cells by upregulation of lineage markers, downregulation of stem/progenitor markers and reduced sphere formation capability.

### Extracellular Matrix Proteins Alter the Expression of Differentiation Markers in GSCs

**Fig. 3:**
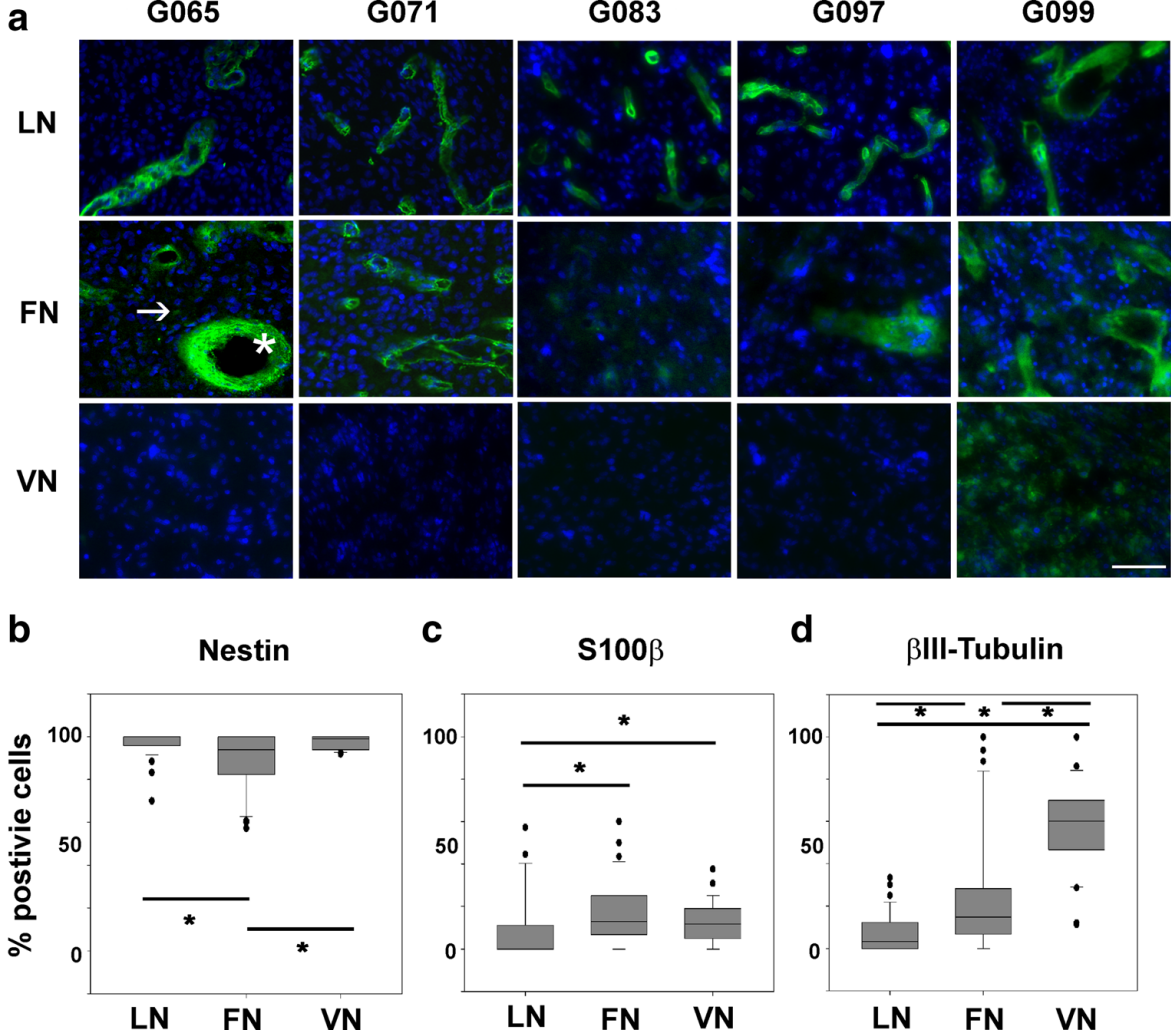
Extracellular matrix proteins alter the expression of differentiation markers in GSCs. **a** Immunostaining of five tissue samples (G065, G071, G083, G097, G099) for laminin (LN), fibronectin (FN) and vitronectin (VN) in *green*, nuclei stained with DAPI (*blue*); the *star* indicates FN expression in distinct regions; the *arrow* indicates diffuse FN in tissue. *Scale bar* = 50 μm. **b**–**d** Percentage of GSCs positive for **b** nestin, median values LN 95.9 %, FN 82.4 % and VN 94.0 % , **c** S100β, median values LN 0.0 %, FN 12.8 % and VN 11.5 % and **d** βIII-tubulin, median values LN 3.3 %, FN 14.7 % and VN 60.3 % from G100 GSC line cultured on LN, FN or VN for 14 days and immunostained for the three markers. *N* = 3 wells per condition; *P < 0.05 using Kruskal-Wallis followed by Tukey’s posthoc test

We then wanted to investigate candidate migratory substrates available in the tumourigenic niche and to test the effect of ADAM10 and ADAM17 inhibition on migration on these candidate substrates. We chose to focus on the basement membrane proteins laminin and fibronectin, and vitronectin which has been shown to be expressed at the leading edge of the tumour [20], to elucidate their roles in GSC migration and differentiation. Resected tissue from five patients were analysed (Fig. 3a). Both laminin and fibronectin were detected in all five samples with laminin expression being solely in distinct regions; fibronectin was also observed in distinct regions (Fig. 3a, star) as well as diffusely throughout the tissue (Fig. 3a, arrow), whereas vitronectin was only expressed in 1/5 tissue samples. To investigate if different ECMs could affect the phenotype of the GSCs, we cultured isolated GSCs as monolayers on different ECMs for 14 days and found that the ECMs altered expression of stem/lineage markers. Nearly 100 % of the cultured GSCs expressed the stem/progenitor cell marker nestin on all ECMs; percentage on fibronectin was significantly lower than on laminin and vitronectin (Fig. 3b). For the astrocyte marker S100β, expression was low in general and was significantly increased on both fibronectin and vitronectin compared to laminin (Fig. 3c); whereas for the neuronal marker βIII-tubulin, expression was low in cells on laminin and fibronectin but higher on vitronectin and significantly different between all three ECMs (Fig. 3d). In summary, ECM proteins can affect cell differentiated status; cells are less differentiated on laminin and more differentiated on vitronectin and to a smaller extent on fibronectin. We therefore wanted to assess the effect of ECM proteins on GSC migration.

### ADAM10 or ADAM17 Inhibition Increases Cell Migration out of Tumourspheres on Three Different ECMs

**Fig. 4:**
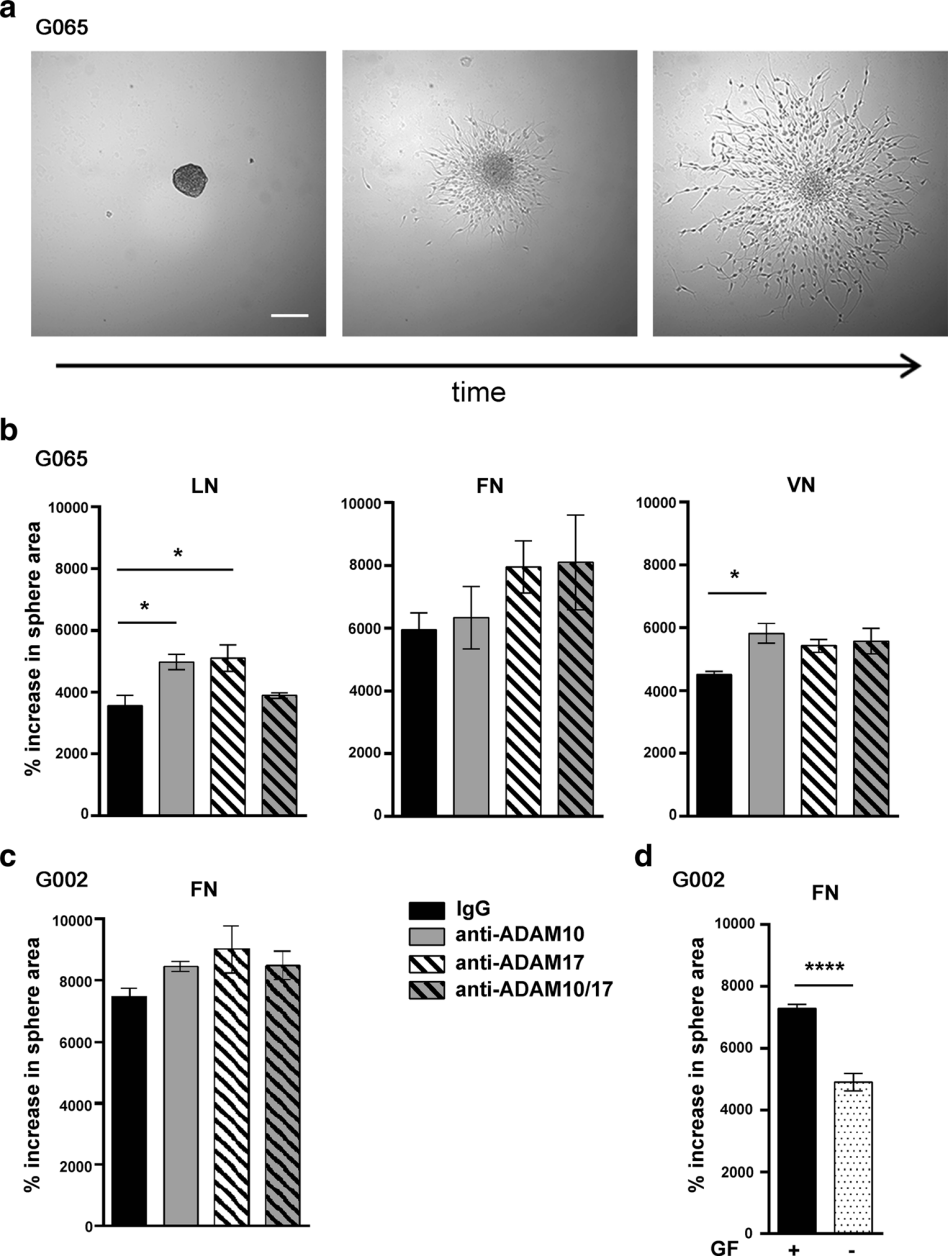
ADAM10 or ADAM17 inhibition increases cell migration out of tumourspheres on three different ECMs. Tumoursphere spreading assay: Individual tumourspheres (100 μm in diameter) were incubated with 1 μM Ara-C and either IgG (2 μg/ml), anti-ADAM10 (0.2 μg/ml), anti-ADAM17 (2 μg/ml) or anti-ADAM10 and ADAM17 combined. Spheres were imaged five times at regular intervals between *t* = 0 h and *t* = 45 h; sphere area was measured using Image J. Cell migration out of the sphere was calculated as ((*t* = 45 h − *t* = 0 h)/*t* = 0 h) × 100 to give percent increase in sphere area. **a** Phase contrast images of sphere migration from a single G065 sphere on LN at 0 h, 20 h and 45 h, *scale bar* = 100 μm. **b** Cell migration out of G065 spheres was tested on wells pre-coated with three ECMs; laminin (LN), fibronectin (FN) or vitronectin (VN) at 25 μg/ml. *N* = 4–6 spheres per condition; *P < 0.05 using one-way ANOVA followed by Bonferroni’s posthoc test. **c** Cell migration out of G002 spheres was tested on wells pre-coated with FN (25 μg/ml), *N* = 4–6 spheres per condition; *P* > 0.05 using one-way ANOVA. **d** Cell migration out of G002 spheres on fibronectin in the absence or presence of growth factors (GF) when treated with IgG only. *N* = 5–6 spheres per condition; **P < 0.0001 using unpaired *t* test

To analyse migration in a 3D model that may be more relevant to the in vivo situation, we plated GSC-derived tumourspheres onto ECM protein-coated wells containing the anti-mitotic compound, cytosine β-*D*-arabinofuranoside (Ara-C), to block cell proliferation as well as blocking antibodies or IgG control. We found that ADAM10 and ADAM17 inhibition had slightly different effects depending on which ECM protein cells were plated on, but overall, they increased migration of GSCs out of tumourspheres, measured by an increased area of the spheres (Fig. 4a–c). The mitogenic growth factor mix used in culture medium was also included in the migration media as it significantly increased cell migration out of the sphere (Fig. 4d). This shows that inhibition of ADAM10 and ADAM17 increases cell migration out of tumourspheres plated on laminin, fibronectin and vitronectin.

**Fig. 5:**
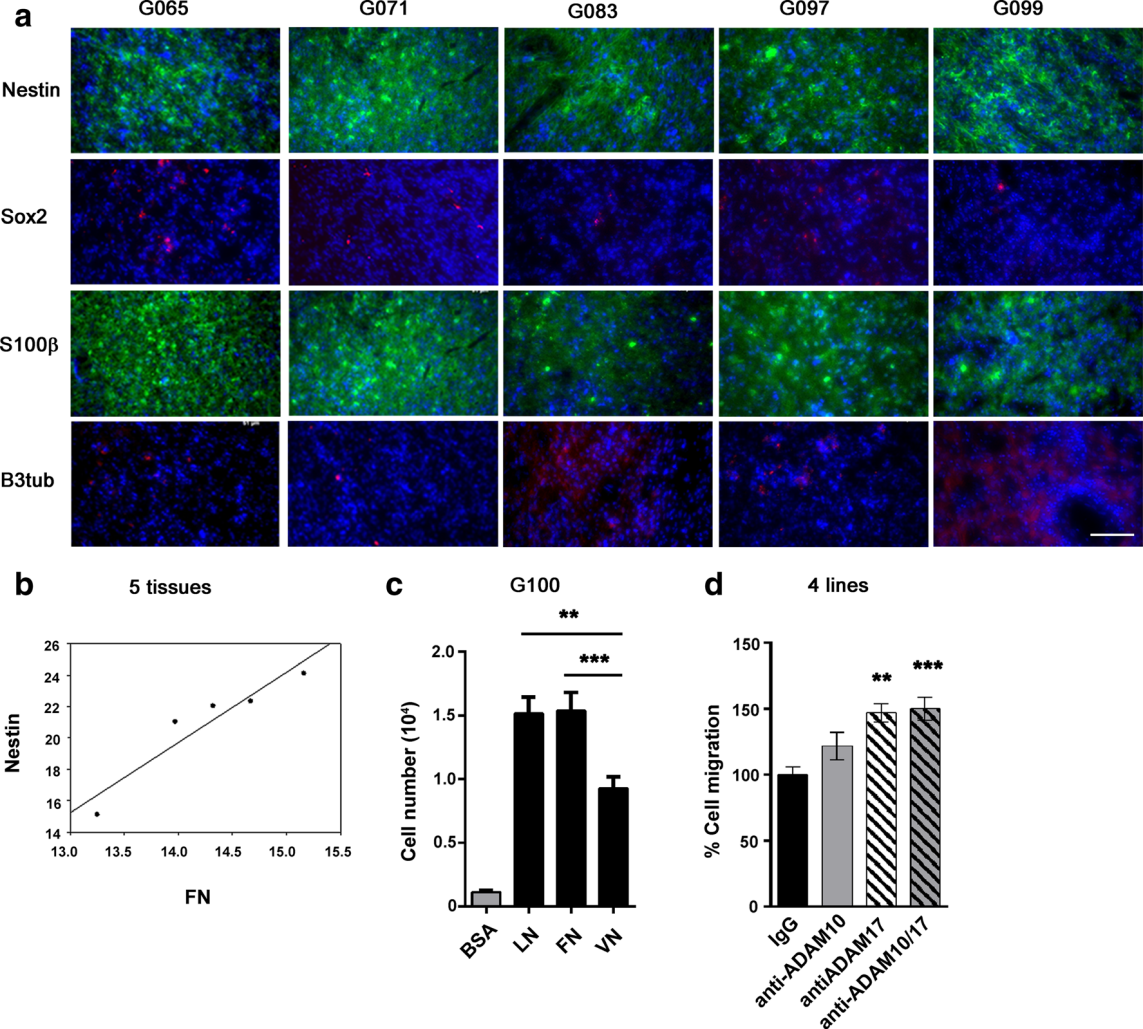
ADAM10 and ADAM17 inhibition increases GSC migration towards fibronectin. **a** Immunostaining of G065, G071, G083, G097 and G099 tissue samples for stem/progenitor cell marker nestin (*green*), stem cell marker Sox2 (*red*), astrocyte marker S100β (*green*), neuronal marker βIII-tubulin (*red*), nuclei stained with DAPI (*blue*), *scale bar* = 50 μm. **b** Correlation between nestin and fibronectin (FN) intensity expression in five tissues, G065, G071, G083, G097 and G099 (Pearson product moment correlation test, *r* = 0.943, *P* = 0.016, Sigma plot). **c** Haptotactic migration of G100 GSCs through ECM protein-coated transwell over 24 h. *N* = 4 wells per condition; **P < 0.01, ***P < 0.001 using one-way ANOVA followed by Tukey’s posthoc test. **d** Haptotactic migration of four GSC lines (G002, G100, G109, G112) over 24 h through fibronectin in absence of growth factors. Cells were treated with IgG isotype control (2 μg/ml), anti-ADAM10 (0.2 μg/ml), anti-ADAM17 (2 μg/ml) or anti-ADAM10 and ADAM17 combined. *N* = 4 wells per condition and per cell line; **P < 0.01, ***P < 0.001 using one-way ANOVA followed by Tukey’s posthoc test

To see if this in vitro observation correlated with the in vivo situation, we looked for correlations between expression of individual ECMs and cell phenotype in five different tissue samples. Again, we used nestin, Sox2, βIII-tubulin and S100β to assess the cell differentiation state and found all tissues exhibited relatively high numbers of cells expressing nestin and S100β, but low numbers of cells expressed βIII-tubulin and Sox2, as expected for this type of tumour (Fig. 5a). We then compared this to the expression of laminin, fibronectin and vitronectin in the same five tissue samples and found a significant correlation between fibronectin and nestin (Fig. 5b). This suggests that ECM and fibronectin in particular might be involved in GSC migration.

### ADAM10 and ADAM17 Inhibition Increases GSC Migration Towards Fibronectin

Based on its expression pattern, effect on differentiation markers and correlation with stem/progenitor marker, we focused on fibronectin in further experiments using one of the GSC lines (G100). We first compared the effectiveness of fibronectin against laminin and vitronectin for the ability to induce GSC haptotactic migration by coating the underside of the transwell membrane with laminin, fibronectin or vitronectin. All three ECMs were effective haptotactic stimuli for GSCs, in the absence of mitogens, with laminin and fibronectin being the most effective confirming that fibronectin is a suitable in vitro substrate for GSC migration (Fig. 5c). We then used fibronectin as the migration substrate during ADAM10 and ADAM17 inhibition and found that fibronectin mediated directional migration of GSCs was increased during ADAM10 and ADAM17 blockade (Fig. 5d). We have shown here that ADAM10 and ADAM17 inhibition increase GSC migration through fibronectin.

### GSC Adhesion to Fibronectin Is Mediated by Integrin α5β1

**Fig. 6:**
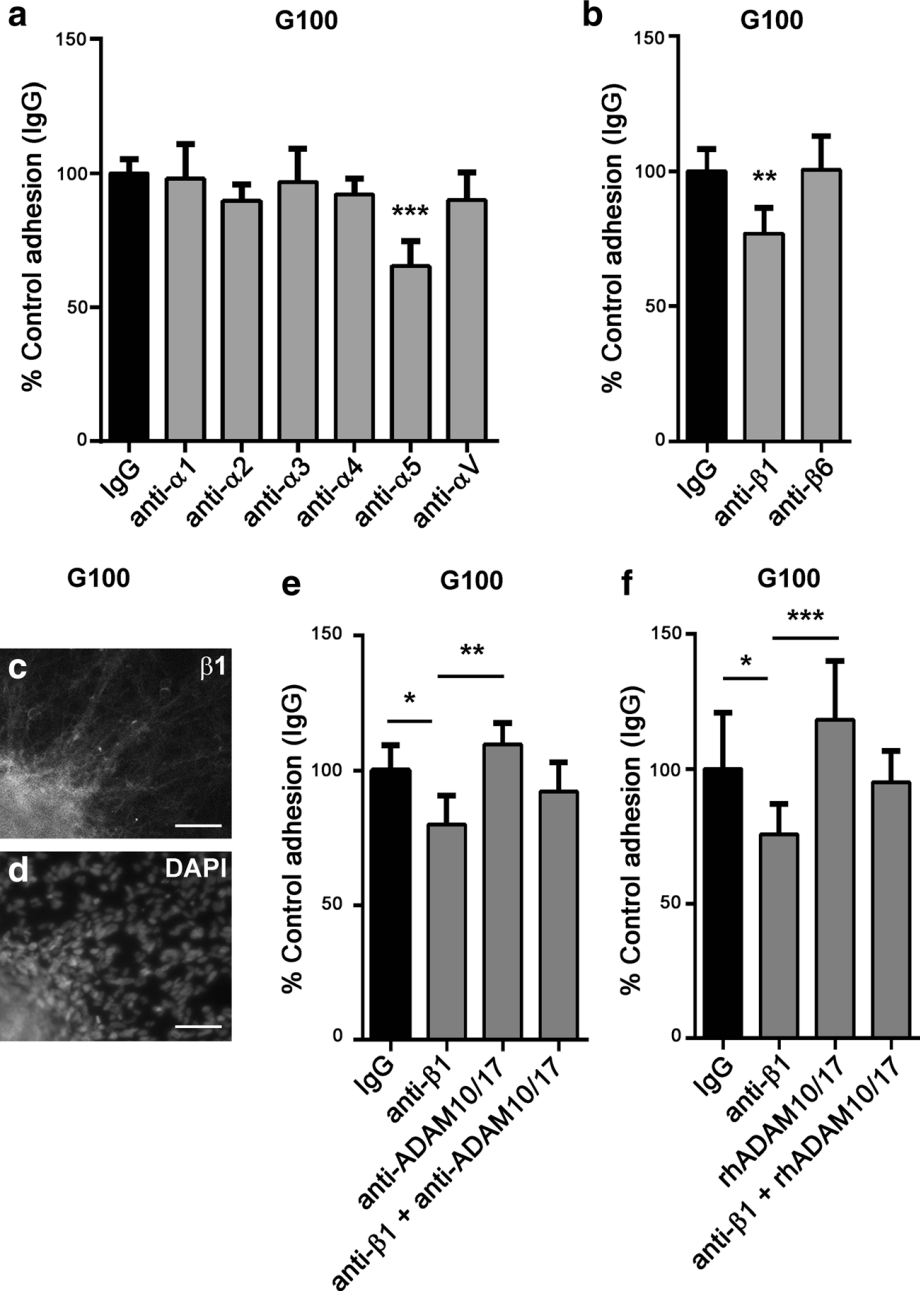
GSC adhesion to fibronectin is mediated by integrin α5β1 and interaction with ADAM10 and ADAM17. Adhesion of GSCs to fibronectin over 1.5 h. Dissociated cells from G100 GSC line were incubated with antibodies blocking α (**a**) or β (**b**) integrins and IgG isotype control. *N* = 6–8 wells per condition; **P < 0.01, ***P < 0.001 compared to IgG control, using one-way ANOVA followed by Tukey’s posthoc test. Expression of β1 integrin (**c**) and DAPI (**d**) in G100 tumoursphere adhered to fibronectin; *scale bar* =30 μm. (**e**–**f**) Adhesion of G100 GSCs to fibronectin over 1.5 h. Cells were treated with β1-blocking antibody with either ADAM10 and ADAM17 blocking antibodies (**e**) or recombinant human (rh) ADAM10 and ADAM17 (**f**). *N* = 4–5 wells per condition (**e**), *N* = 8 wells per condition (**f**); *P < 0.05, **P < 0.01, ***P < 0.001 using one-way ANOVA followed by Tukey’s posthoc test

To migrate, cells must adhere to surrounding substrates and this process involves integrins. We screened blocking antibodies to α and β integrin subunits and found that α5β1, the classical fibronectin receptor, mediates adhesion of GSCs to fibronectin (Fig. 6a, b). β1 integrin is expressed in GSCs (Fig. 6c, d) in which the blocking antibody consistently reduced cell adhesion by 25 % (Fig. 6b, e, f). We further investigated GSC adhesion to fibronectin and found that ADAM10 and ADAM17 inhibition or exogenous recombinant ADAM10 and ADAM17 had no significant effect on this process (Fig. 6e, *P* = 0.1193, Fig. 6F, *P* = 0.1110), but when adding anti-ADAM10 and ADAM17 or exogenous recombinant human (rh) ADAM10 and ADAM17, β1 integrin inhibition no longer significantly reduces cell adhesion (Fig. 6e, f). This suggests that β1 integrin interacts with ADAM10 and ADAM17 and that its effect is blocked by ADAM10 and ADAM17 blocking antibodies or exogenous ADAM10 and ADAM17.

## Discussion

A major hurdle in glioblastoma treatment is the difficulty of complete surgical resection due to the diffuse nature of the tumour, which invariably results in tumour recurrence. Altering cell migration/invasion, especially in the tumour initiating cells, would act to constrain the tumour mass and/or remove the tumour initiating cells from their niche, thereby increasing the efficacy of resection. We investigated whether ADAM10 and ADAM17 have a role in this process.

Cell migration in the brain is a carefully orchestrated process involving precision-timed attractant and repellent signalling along with many structural components. We used the transwell migration system to assess directional migration towards different chemoattractants, and the greatest stimulus was provided by the combination of growth factors which are included in the GSC maintenance media.

When using growth factors as a stimulus, anti-ADAM10 or anti-ADAM17 significantly increased GSC migration, but this was not true for NSCs suggesting an inherent difference in cellular response to metalloproteinase inhibition between tumour and non-tumour stem cells. This suggests that ADAM10 and ADAM17 are important components of the tumourigenic niche which function to retain cancer stem cells in this niche, promoting self-renewal and tumour recurrence. GSCs have not only increased expression of ADAMs compared to NSCs [5] but also elevated receptor tyrosine kinases whose ligands are shed by ADAM10 and ADAM17, in particular EGFR, which is often upregulated in glioblastoma [21–24]. Increased ADAM expression can result in greater growth factor and cytokine processing [25–27], conversely in mouse embryonic fibroblasts; knockdown of ADAM10 ablated EGF shedding [12] which suggests that in our experimental settings, EGF processing may also be reduced.

GSCs may have a higher dependence on growth factors than NSCs and so inhibition of ADAM10 and ADAM17 to reduce endogenous growth factors could stimulate migration of GSCs to an area of higher growth factor concentration (i.e. the lower transwell chamber). This theory would also explain the migration of cells out of tumourspheres where the removal of growth factors from the surrounding media was found to significantly slowdown migration. Again ADAM10 and ADAM17 inhibition, which would reduce local growth factor concentrations, promoted the migration of GSCs into the growth factor containing media. Mitogenic growth factors such as EGF would be expected to be vital components of the tumourigenic niche acting to maintain self-renewal and also as chemoattractants to retain or even home stem cells to the niche.

As ADAM10 and ADAM17 are overexpressed in glioblastoma where they can act to increase the EGF concentration in the niche then anti-ADAM10 and ADAM17 treatment may mediate migration out of the niche by reducing local EGF concentrations.

Chen et al. analysed migration in the human glioblastoma cell line U87 cultured in serum using matrigel-coated transwell and showed a decrease of migration when ADAM17 was inhibited [28]. Similarly, Zheng et al. showed a decrease in invasive properties of the human glioblastoma cell line U87 over 24 h of ADAM17 inhibition, using matrigel-coated transwell with serum in the lower chamber and analysing the number of cells on the underside of the membrane [29]. We found that many cells had detached from the underside of the transwell membrane into the lower chamber and so counted the total cell number in the lower chamber which may account for the difference in results. Moreover, our cells are low passage sphere-forming cell populations from high grade gliomas, cultured without serum, and so are likely to exhibit a different phenotype from the U87 cells cultured with differentiation promoting serum.

Over 24 h, ADAM10 and ADAM17 inhibition increased GSC migration and the migrated cells had started to differentiate and loose the stem cell phenotype. ADAM10 and ADAM17 can influence cell fate through Notch processing [30, 31], yet ADAM inhibition did not significantly alter the expression of differentiation markers between migrated cells and non-migrated cells (data not shown); this may be due to the short time period in which the cells were exposed to the ADAM10 and ADAM17 blocking antibodies. Indeed, we and others found that ADAM10 and ADAM17 inhibition increased neuronal marker βIII-tubulin expression over a week in differentiation culture conditions and promoted asymmetrical division giving rise to neuronal progenitors [5, 31, 32]. A negative correlation has been found in glioblastoma between the genetic signature of the epithelial-to-mesenchymal transition (EMT) and that of CD133 [33], suggesting that ADAM10 and ADAM17 inhibition might activate the transition, differentiating the cells and increasing cell migration and invasion.

In further assessment of GSC differentiation, we investigated the contribution of ECMs to the stem cell phenotype. Laminin is a useful tool for isolation of GSCs from glioblastoma tissues [18]; we have shown that low passage GSCs plated on laminin exhibit high expression of stem/progenitor cell marker with minimal differentiation along glial and neuronal lineages at day 14 suggesting laminin is capable of maintaining the stem cell phenotype. Laminin also maintains cells in an undifferentiated state better than fibronectin and vitronectin, the latter being the most permissive to cell differentiation.

All three ECMs are effective haptotactic substrates for GSC migration, but are they all involved in glioblastoma invasion? Laminin is a component of the perivascular niche [34], but it is unlikely to be the substrate for migration throughout the brain as its expression is restricted to basement membranes [35, 36]). Vitronectin is a possible candidate as it is expressed at the leading edge of the tumour [20] but is unlikely to be the only one, as we found, it is only expressed in 20 % of our samples. In comparison, as fibronectin is found around blood vessels and diffuse throughout the tissue [37], it provides the best migratory route from the perivascular niche out into the brain parenchyma. Moreover, glioblastoma cells have been found to migrate along fibronectin positive tracts in the rat brain [38]. We show here a positive correlation between stem/progenitor cells and fibronectin in high grade glioma tissues, as well as an increase in GSC migration through fibronectin during ADAM10 and ADAM17 inhibition, as previously shown with renal cancer cells [39]. To better understand the effect of the blocking antibodies on this migration, we investigated the disintegrin function of ADAM10 and ADAM17 during GSC adhesion to fibronectin. The disintegrin domain of ADAM10 and ADAM17 is not homologous to the other ADAM family members and is likely to bind different proteins.

Integrin subunit screening detected that α5β1 integrins are required for GSC adhesion to fibronectin; ADAM17 is known to bind to this integrin in vitro [40, 41], and we showed that blocking α5 or β1 decreased adhesion of GSC to fibronectin. Addition of exogenous rhADAM10 and ADAM17 had the same effect as ADAM10 and ADAM17 blocking antibodies suggesting that the proteinase function of ADAMs is not involved in cell adhesion. Any effect is more likely due to direct interaction between ADAMs and integrins. When the recombinant protein or blocking antibodies are present, β1 integrin blockade no longer decreases cell adhesion suggesting there may be disruption of ADAM interaction with integrins either by ADAM10 and ADAM17 blocking antibodies preventing interaction or by rhADAM10 and ADAM17 blocking binding sites on the integrin. Even though ADAM inhibition did not alter GSC adhesion to fibronectin over 1.5 h, migration is equilibrium between adhesion and detachment, and an effect through the disintegrin domain cannot be excluded and could still account for the increased cell migration observed during ADAM10 and ADAM17 blockade.

Although TAPI-2 is a commonly used inhibitor of ADAM17, it is not specific to ADAM17 and can also inhibit matrix metalloproteinases or MMPs. We therefore chose blocking antibodies to specifically inhibit ADAM10 or ADAM17. We confirmed the efficacy of anti-ADAM17 to inhibit ADAM17 proteinase activity using purified recombinant ADAM17 and non-purified GSC lysates, at similar levels as TAPI-2. Although the anti-ADAM17 significantly decreased ADAM17 activity in both situations, it was less efficient in the GSC lysates suggesting that ADAM17 collected from GSCs might bind other proteins and the blocking antibody may not efficiently compete for the binding site.

We have shown for the first time that growth factors and ECMs are effective stimuli for GSC migration and that they are likely to be important components of the tumourigenic niche in the brain where they can support the stem cell phenotype. As ADAM inhibition increases GSC migration, ADAM10 and ADAM17 may also be key components of this niche where they act to retain cells in the tumourigenic environment in an undifferentiated state. Although blocking ADAM10 and ADAM17 may increase GSC invasion in vivo, if the migrating cells are differentiating, they will lose stem cell characteristics; this will be compounded by the fact they are also moving away from the niche environment that supports stemness, creating a feed forward process. In combination with ADAM10 and ADAM17 inhibition decreasing GSC proliferation, these new therapeutic targets may provide a mechanism for depletion of the tumourigenic stem cell pool that seeds the tumour whilst sparing native neural stem cells.
